# Evaluation of Clear Cell, Papillary, and Chromophobe Renal Cell Carcinoma Metastasis Sites and Association With Survival

**DOI:** 10.1001/jamanetworkopen.2020.21869

**Published:** 2021-01-21

**Authors:** Shaan Dudani, Guillermo de Velasco, J. Connor Wells, Chun Loo Gan, Frede Donskov, Camillo Porta, Anna Fraccon, Felice Pasini, Jae Lyun Lee, Aaron Hansen, Georg A. Bjarnason, Benoit Beuselinck, Sumanta K. Pal, Takeshi Yuasa, Nils Kroeger, Ravindran Kanesvaran, M. Neil Reaume, Christina Canil, Toni K. Choueiri, Daniel Y. C. Heng

**Affiliations:** 1 Tom Baker Cancer Centre, University of Calgary, Calgary, Alberta, Canada; 2 Department of Medical Oncology, University Hospital 12 de Octubre, Madrid, Spain; 3 Department of Oncology,Aarhus University Hospital, Aarhus, Denmark; 4 Department of Internal Medicine, University of Pavia, Pavia, Italy; 5 Now with Department of Oncology, University of Bari Aldo Moro, Bari, Italy; 6 CDC Pererzoli, Peschiera del Garda, Italy; 7 Oncologia Medica Ospedale Santa Maria della Misericordia, Rovigo, Italy; 8 Asan Medical Center, University of Ulsan College of Medicine, Seoul, South Korea; 9 Division of Medical Oncology and Hematology, Princess Margaret Cancer Centre, Toronto, Ontario, Canada; 10 Division of Medical Oncology, Sunnybrook Research Institute, Toronto, Ontario, Canada; 11 Leuven Cancer Institute, University Hospitals Leuven, Leuven, Belgium; 12 City of Hope Comprehensive Cancer Center, Duarte, California; 13 Cancer Institute Hospital of Japanese Foundation for Cancer Research, Tokyo, Japan; 14 Department of Urology, University Medicine Greifswald, Greifswald, Germany; 15 Department of Medical Oncology, National Cancer Centre Singapore, Singapore; 16 The Ottawa Hospital Cancer Centre, University of Ottawa, Ottawa, Ontario, Canada; 17 Dana-Farber Cancer Institute, Brigham and Women’s Hospital, Harvard Medical School, Boston, Massachusetts

## Abstract

**Importance:**

There exists considerable biological and clinical variability between histologic variants of metastatic renal cell carcinoma (mRCC). Data reporting on patterns of metastasis in histologic variants of mRCC are sparse.

**Objective:**

To characterize sites of metastasis and their association with survival across the 3 most common histologic variants of mRCC: clear cell (ccRCC), papillary (pRCC), and chromophobe (chrRCC).

**Design, Setting, and Participants:**

In this multicenter, international cohort study, the International mRCC Database Consortium (IMDC) database was used to identify consecutive patients starting systemic therapy for mRCC between 2002 and 2019. Patients with mixed histologic subtype were excluded. Statistical analysis was performed from February to June 2020.

**Exposures:**

Data regarding histologic subtype and sites of metastatic involvement at the time of first systemic therapy initiation were collected.

**Main Outcomes and Measures:**

The primary outcomes were prevalence of metastatic site involvement and overall survival (OS) from time of systemic therapy initiation. Patients with multiple sites of metastatic involvement were included in analyses of all groups to which they had metastases.

**Results:**

A total of 10 105 patients were eligible for analysis. Median (interquartile range) age at diagnosis was 60 (53-67) years, 7310 (72.4%) were men and 8526 (84.5%) underwent nephrectomy. Of these, 9252 (92%) had ccRCC, 667 (7%) had pRCC, and 186 (2%) had chrRCC. The median number of sites of metastasis was 2 (range, 0-7). In ccRCC, the most common sites of metastasis were lung (70%; 6189 of 8804 patients [448 missing]), lymph nodes (45%; 3874 of 8655 patients [597 missing]), bone (32%; 2847 of 8817 patients [435 missing]), liver (18%; 1560 of 8804 [448 missing]), and adrenal gland (10%; 678 of 6673 patients [2579 missing]). Sites of metastasis varied between subtypes. Lung, adrenal, brain, and pancreatic metastases were more frequent in ccRCC, lymph node involvement was more common in pRCC, and liver metastases were more frequent in chrRCC. Median OS for ccRCC varied by site of metastatic involvement, ranging between 16 months (95% CI, 13.7-18.8 months) for the pleura and 50 months (95% CI, 41.1-55.5 months) for the pancreas. Compared with ccRCC, patients with pRCC tended to have lower OS, regardless of metastatic site.

**Conclusions and Relevance:**

Sites of metastatic involvement differ according to histologic subtype in mRCC and are associated with OS. These data highlight the clinical and biological variability between histologic subtypes of mRCC. Patterns of metastatic spread may reflect differences in underlying disease biology. Further work to investigate differences in immune, molecular, and genetic profiles between metastatic sites and histologic subtypes is encouraged.

## Key Points

**Question:**

In metastatic renal cell carcinoma (RCC), do patterns of metastasis differ between histologic subtypes and are sites of metastasis associated with survival?

**Findings:**

In this cohort studyincluding 10 105 patients, patterns of metastasis varied significantly between metastatic clear cell RCC, papillary RCC, and chromophobe RCC. Sites of metastasis were associated with survival in all histologic subtypes.

**Meaning:**

These data highlight the clinical and biological variability between metastatic RCC histologic subtypes and suggest that patterns of metastasis may reflect differences in underlying disease biology.

## Teaser

This cohort study evaluates the prevalence of metastasis sites for clear cell, papillary, and chromophobe renal cell carcinoma (RCC) and examines the association between site of metastasis and overall survival.

## Introduction

Renal cell carcinomas (RCC) comprise a heterogeneous group of malignant neoplasms arising from the nephron. With more than a dozen recognized histologic variants of renal cell tumors,^1^ RCCs may vary widely in their genetic, pathologic, and clinical characteristics. The 3 most common histologic variants of RCC include clear cell RCC (ccRCC), papillary RCC (pRCC), and chromophobe RCC (chrRCC), representing 75% to 85%, 10% to 15% and 5% to 10% of all kidney cancers, respectively.^2,3^ Comprehensive molecular and genetic analyses conducted through The Cancer Genome Atlas (TCGA) program have demonstrated that these subtypes of RCC are biologically distinct.^4,5,6^ Expectedly, the clinical behavior of RCC subtypes is similarly heterogeneous, which is likely a result of the differences in underlying disease biology between histologic variants.^7,8,9,10,11^

One manifestation of the divergent biological underpinnings and ensuing clinical behavior includes the pattern of disease spread in patients with metastatic RCC (mRCC). The most common sites of metastatic involvement in ccRCC are well characterized and include the lung, lymph nodes, bone, and liver.^12,13^ However, the rates of involvement for less common metastatic sites are not well described for this subtype. In addition, sites of metastasis in pRCC and chrRCC have not been as well characterized in the literature, and how the pattern of spread in these histologic variants compares with ccRCC is unclear.

Because sites of metastatic involvement may reflect differences in underlying disease biology, the clinical behavior of mRCC may vary on the basis of the pattern of organ involvement, even within a single histologic subtype. Indeed, it has been reported in select series that patients with mRCC that has metastasized to endocrine organs, such as the pancreas, may have extraordinary clinical outcomes, which may be a result of favorable underlying disease biology.^14,15^ Alternatively, metastases to other organs, such as the liver, bone, and/or brain, are associated with poor outcomes in patients with mRCC.^13,16,17^

Using the International mRCC Database Consortium (IMDC) clinical database—a large international database consisting of more than 11 000 patients from more than 40 institutions worldwide—we sought to characterize and compare the frequency of metastatic site involvement across the 3 major histologic subtypes of RCC and to assess for associations between site of metastatic involvement and survival.

## Methods

### Study Design and Patient Selection

Using the IMDC database, we performed an analysis of consecutive patients with mRCC from 40 international centers. Data were collected from hospital and pharmacy records between January 1, 2005 and December 31, 2019, using uniform database software and templates. All participating centers received approval from the local research ethics board prior to initiation of data collection. Waivers of consent were approved by the local research ethics boards of all participating institutions in order to facilitate maximal capture of the local patient populations and minimize bias. All patient data was deidentified. Results are presented in accordance with the Strengthening the Reporting of Observational Studies in Epidemiology (STROBE) reporting guideline.

All patients with metastatic ccRCC, pRCC, and chrRCC who began systemic therapy between 2002 and 2019 were included. Sites of metastatic involvement known at the time of first systemic therapy initiation for metastatic disease were collected. Patients with mixed histologic profile (ie, 2 or more histologic subtypes on histopathologic evaluation) were excluded.

### Outcome Measurements

Data regarding patient demographic characteristics, baseline characteristics, IMDC risk factors (hemoglobin less than the lower limit of normal, platelet count greater than the upper limit of normal, neutrophil count greater than the upper limit of normal, corrected calcium greater than the upper limit of normal, Karnofsky Performance Status <80%, and time from diagnosis to treatment < 1 year),^18^ tumor and treatment details, sites of metastatic involvement, and survival were extracted from the IMDC. Outcome measures of interest were prevalence of metastatic site involvement and overall survival (OS). OS was calculated from the time of initiation of first-line systemic therapy to death from any cause or censored at the time of last follow-up.

### Statistical Analysis

Patient demographic characteristics and baseline characteristics are described using proportions (%) for categorical variables and medians (range or interquartile range [IQR]) for continuous variables. Differences in categorical and continuous variables were assessed using χ^2^ testing and Kruskal-Wallis testing, respectively. OS was evaluated using the Kaplan-Meier method. χ^2^ testing and log-rank testing were used to assess for differences in sites of metastasis and OS, respectively. Hazard ratios (HRs) were calculated using multivariable Cox regression analyses and adjusted to control for imbalances in individual IMDC risk factors. All OS HRs are reported comparing involved vs noninvolved sites of metastasis (HR > 1 denotes worse OS). Patients with multiple sites of metastatic involvement were included in analyses of all groups to which they had metastases.

*P* <.05 was considered significant. All statistical testing was 2-sided. The case deletion method was used when missing data were encountered. SAS statistical software version 9.4 (SAS Institute) was used to perform statistical analyses from February to June 2020.

## Results

### Patients

Of 11 514 total patients, 10 105 (88%) were eligible for analysis and had available data. Of these, median (IQR) age at diagnosis was 60 (53-67) years, 7310 (72.4%) were men, 8526 (84.5%) underwent nephrectomy, and 1034 (12.6%) had sarcomatoid features. There were 9252 patients with ccRCC (92%), 667 patients with pRCC (7%) and 186 patients with chrRCC (2%). Patients with chrRCC were less likely to be male (54%; 101 of 186 patients) and more likely to have sarcomatoid features (21%; 37 of 176 patients). Most patients were treated in North America (50%; 5072 patients) or Europe (35%; 3515 patients). Across the entire cohort, IMDC risk groups were: 19% favorable (1530 patients), 57% intermediate (4621 patients), and 25% poor (2002 patients). Patients with pRCC were least likely to have IMDC favorable-risk disease (14%; 71 patients) and most likely to have IMDC poor-risk disease (29%; 150 patients). Most patients received VEGF targeted therapy in the first line (88%; 8895 patients). Patients with pRCC and chrRCC were less likely to receive first-line VEGF targeted therapy (71% [475 of 667 patients] and 72% [134 of 186 patients], respectively). Baseline characteristics are summarized in Table 1.

**Table 1.** Baseline Characteristics

Characteristic	Patients, No./total (%)				*P* value^a^
	Total (N = 10 105)	ccRCC (n = 9252)	pRCC (n = 667)	chrRCC (n = 186)	
Age, median (IQR), y	60 (53-67)	60 (53-67)	61 (51-69)	58 (48-65)	.002
Sites of metastasis, median (range), No.	2 (0-7)	2 (0-7)	2 (0-6)	2 (0-4)	.001
Men	7310/10 104 (72.4)	6712/9251 (72.6)	497/667 (74.5)	101/186 (54.3)	<.001
Sarcomatoid features	1034/8223 (12.6)	944/7523 (12.6)	53/524 (10.1)	37/176 (21.0)	<.001
Nephrectomy	8526/10 094 (84.5)	7809/9244 (84.5)	545/664 (82.0)	172/186 (92.5)	.003
Region					
Asia	1180/10 105 (11.7)	1072/9252 (11.6)	93/667 (13.9)	15/186 (8.1)	<.001
Europe	3515/10 105 (34.8)	3299/9252 (35.7)	162/667 (24.3)	54/186 (29.0)	
North America	5072/10 105 (50.2)	4564/9252 (49.3)	397/667 (59.5)	111/186 (59.7)	
Oceania	338/10 105 (3.3)	317/9252 (3.4)	15/667 (2.2)	6/186 (3.2)	
Year of systemic therapy start					
2002-2005	492/10 105 (4.9)	460/9252 (5.0)	24/667 (3.6)	8/186 (4.3)	.02
2006-2010	3896/10 105 (38.5)	3599/9252 (38.9)	244/667 (36.6)	53/186 (28.5)	
2011-2015	3749/10 105 (37.1)	3416/9252 (36.9)	257/667 (38.5)	76/186 (40.9)	
2015-2019	1968/10 105 (19.5)	1777/9252 (19.2)	142/667 (21.3)	49/186 (26.3)	
IMDC risk groups					
Favorable	1530/8153 (18.8)	1422/7489 (19.0)	71/514 (13.8)	37/150 (24.7)	.004
Intermediate	4621/8153 (56.7)	4251/7489 (56.8)	293/514 (57.0)	77/150 (51.3)	
Poor	2002/8153 (24.6)	1816/7489 (24.3)	150/514 (29.2)	36/150 (24.0)	
First-line treatments					
VEGF targeted agent	8895/10 105 (88.0)	8286/9252 (89.6)	475/667 (71.2)	134/186 (72.0)	<.001
mTOR targeted agent	451/10 105 (4.5)	312/9252 (3.4)	111/667 (16.7)	28/186 (15.1)	
ICI based regimen	624/10 105 (6.1)	575/9252 (6.2)	29/667 (4.4)	20/186 (10.8)	
Other	135/10 105 (1.3)	79/9252 (0.9)	52/667 (7.8)	4/186 (2.2)	

Abbreviations: ccRCC, clear cell renal cell carcinoma; chrRCC, chromophobe renal cell carcinoma; ICI, immune checkpoint inhibitor; IMDC, International Metastatic Renal Cell Carcinoma Database Consortium; IQR, interquartile range; mTOR, mammalian target of rapamycin; pRCC, papillary renal cell carcinoma; VEGF, vascular endothelial growth factor.

^a^ χ^2^ test across all 3 groups.

### Sites of Metastasis

The median number of sites of metastasis was 2 (range, 0-7 sites). (Patients with 0 documented sites of metastasis may have had recurrent and/or metastatic disease to areas not captured within the IMDC database [eg, locoregional recurrence, skin and soft tissue, parotid gland, other atypical sites].) The most common sites of metastasis across the entire cohort were lung, lymph nodes, bone, liver, adrenal, and brain. Less frequent sites of metastasis (<5%) included pancreas, pleura, peritoneum, spleen, thyroid, and bowel.

**Figure 1.**
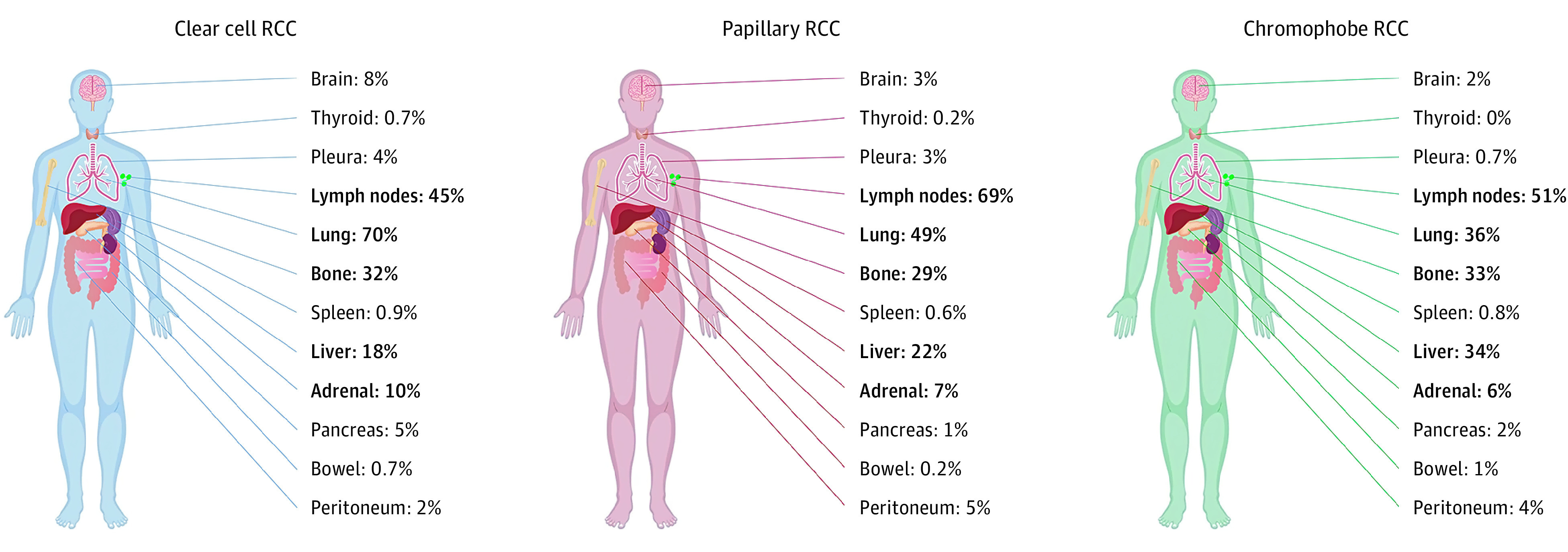
Sites of Renal Cell Carcinoma (RCC) Metastasis by Histologic Subtype The percentage of patients with involved site of metastasis at the time of first systemic therapy initiation for metastatic disease are presented. The 5 most frequent sites of metastasis across all histologic profiles are highlighted in bold type.

Sites of metastasis by histologic variant are shown in Figure 1. Sites of metastasis varied significantly between histologic subtypes (eTable 1 in the Supplement). Lung, adrenal, brain, and pancreatic metastases were more frequent in ccRCC, lymph node and peritoneal metastases were more frequent in pRCC, and liver metastases were more common in chrRCC. Approximately one-third of patients had bone metastases in all 3 histologic subtypes. The rates of brain metastases were 8% in ccRCC, 3% in pRCC, and 2% in chrRCC (Figure 1).

IMDC risk groups by site of metastatic involvement in ccRCC are presented in the eFigure in the Supplement. Patients with metastases to the pancreas and thyroid had the highest rates of favorable-risk disease (34% and 37%, respectively) and lowest rates of poor-risk disease (12% and 17%, respectively). Sites with a tendency toward higher-risk disease (favorable risk <20% and poor risk >30%) were bone, liver, pleura, and bowel.

Patients with metastatic disease involving only 1 site were observed in 3047 patients with ccRCC (33%), 248 patients with pRCC (37%), and 80 patients with chrRCC (43%) (*P* = .001). In ccRCC, the most common sites of solitary metastasis were lung (54%; 1652 of 3036 patients [11 missing]), bone (18%; 557 of 3038 patients [9 missing]), lymph nodes (16%; 481 of 2955 patients [92 missing]), and liver (6%; 193 of 3033 patients [14 missing]). All other sites were less than 3%. The pancreas and brain were the solitary metastatic site in 2.3% (51 of 2186 patients [861 missing]) and 1.4% (43 of 3030 patients [17 missing]), respectively.

The median number of concurrently involved sites for each site of metastasis is shown in eTable 2 in the Supplement. There was no significant association between age at diagnosis of mRCC and number of metastatic sites.

We performed a post hoc exploratory analysis of rates of sarcomatoid differentiation in patients with chrRCC. We found rates of sarcomatoid differentiation among patients with metastatic chrRCC and involvement of lung, lymph nodes, bone and liver to be 38%, 23%, 14% and 18%, respectively.

### Survival

**Figure 2.**
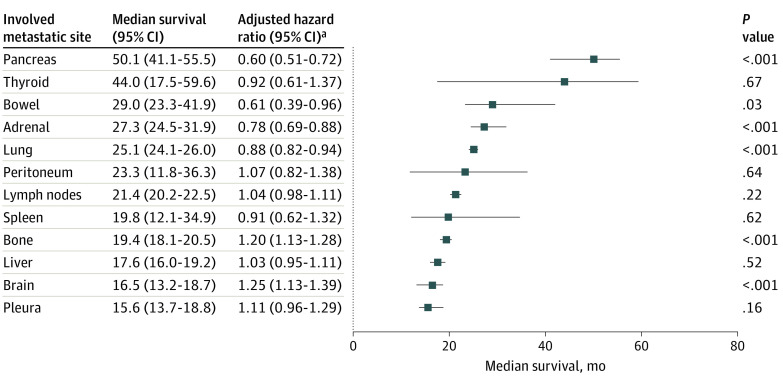
Survival by Site of Metastatic Involvement in Clear Cell Renal Cell Carcinoma (RCC) Survival time is presented in descending order (error bars indicate 95% CIs) for: pancreas (353 patients), thyroid (48 patients), bowel (46 patients), adrenal (678 patients), lung (6189 patients), peritoneum (117 patients), lymph nodes (3874 patients), spleen (55 patients), bone (2847 patients), liver (1560 patients), brain (705 patients), and pleura (295 patients). Overall survival was calculated from time of first systemic therapy initiation for metastatic disease to death from any cause or censored at the time of last follow-up. ^a^Comparing involved vs noninvolved site of metastasis, adjusted by number of International mRCC Database Consortium criteria, number of sites of metastasis, sarcomatoid features, and year started systemic therapy. Hazard ratio greater than 1 denotes worse overall survival.

Survival varied substantially based on site of metastatic involvement. OS results by site of metastatic involvement in patients with ccRCC are illustrated in Figure 2. For these patients, median OS ranged between 16 months (pleura) and 50 months (pancreas). Metastases to liver, brain, and pleura were associated with the shortest median OS times (<18 months).

Because of smaller patient numbers in the pRCC and chrRCC cohorts, OS was calculated for only the 4 most common sites of metastases (lung, lymph nodes, bone, and liver). Compared with patients with ccRCC, patients with pRCC had inferior survival across all sites of metastasis. Conversely, patients with chrRCC generally had prolonged OS times compared with those with ccRCC, with the notable exception of those with lung involvement. Among patients with lung metastases, median survival was inferior for those with chrRCC compared with ccRCC (14.1 months [95% CI, 8.2-23.8 months] vs 25.1 months [24.1-26.0 months]; *P* < .001). The differences in OS did not reach significance in patients with liver metastases. OS results by site of metastasis and histologic profile are shown in Table 2.

**Table 2.** Survival by Histologic Subtype and Site of Metastatic Involvement

Metastatic site	Median survival (95% CI), mo			*P* value^a^
	ccRCC	pRCC	chrRCC	
Lung (n = 6567)	25.1 (24.1-26.0)	15.6 (12.5-19.0)	14.1 (8.2-23.8)	<.001
Lymph nodes (n = 4398)	21.4 (20.2-22.5)	14.3 (12.8-17.2)	28.1 (21.2-36.6)	<.001
Bone (n = 3095)	19.4 (18.1-20.5)	11.0 (9.8-14.1)	26.7 (18.4-35.6)	<.001
Liver (n = 1767)	17.6 (16.0-19.2)	11.8 (9.6-13.9)	26.0 (12.9-36.8)	.07

Abbreviations: ccRCC, clear cell renal cell carcinoma; chrRCC, chromophobe renal cell carcinoma; pRCC, papillary renal cell carcinoma.

^a^ χ^2^ test across all 3 groups.

## Discussion

The results of our cohort study suggest that patterns of metastasis vary considerably among the 3 most common histologic subtypes of RCC and that sites of metastatic involvement are associated with survival. To our knowledge, these represent the largest cohorts to characterize sites of metastasis and report on outcomes specific to metastatic ccRCC, pRCC, and chrRCC.

For all 3 histologic variants, the 5 most common sites of metastatic involvement were the same: lungs, lymph nodes, bone, liver, and adrenals. However, the proportion of patients with metastases to each organ site often differed substantially between subtypes. For example, patients with metastatic ccRCC were nearly twice as likely to have lung metastases than patients with chrRCC, whereas the opposite was true in the case of liver metastases.

Our results are largely consistent with prior data reported for each histologic subtype. Table 3 summarizes rates of metastatic site involvement from a range of prospective and retrospective series of patients with metastatic ccRCC,^12,13,19,20,21^ pRCC,^22,23^ and chrRCC.^24^ There is notable consistency of our data with that of previously reported randomized clinical trials and retrospective studies.

**Table 3.** Studies Reporting on Sites of Metastasis in mRCC

Variable	Patients, No./total (%)										
	ccRCC						pRCC			chrRCC	
	IMDC Cohort 2020	Motzer et al^19^	Rini et al^20^	Rini et al^21^	Chandrasekar et al^a^^13^	Bianchi et al^a^^,^^b^^12^	IMDC Cohort 2020	Escudier et al^22^	Choueiri et al^23^	IMDC Cohort 2020	Ged et al^24^
Sample size, No.	9252	1096	861	915	6610	11 157	667	92	74	186	109
Study type	Retrospective	Prospective	Prospective	Prospective	Retrospective	Retrospective	Retrospective	Prospective	Prospective	Retrospective	Retrospective
Lung	6189/8804 (70.3)	754/1096 (68.8)	621/861 (72.1)	664/915 (72.6)	(51.2)	5039/11 157 (45.2)	312/639 (48.8)	37/92 37 (40.2)	37/74 (50.0)	66/183 (36.1)	37/109 (33.9)
Lymph nodes	3874/8655 (44.8)	514/1096 (46.9)	396/861 (46.0)	429/915 (46.9)	(41.5)	2451/11 157 (21.8)^c^	432/627 (68.9)	75/92 (81.5)	56/74 (75.7)	92/182 (50.6)	47/109 (43.1)
Bone	2847/8817 (32.3)	231/1096 (21.1)	206/861 (23.9)	180/915 (19.7)	(33.5)	3268/11 157 (29.5)	187/640 (29.2)	NA	15/74 (20.3)	61/183 (33.3)	34/109 (31.2)
Liver	1560/8804 (17.8)	206/1096 (18.8)	137/861 (15.9)	160/915 (17.5)	(17.0)	2267/11 157 (20.3)	144/641 (22.5)	15/92 (16.3)	18/74 (24.3)	63/183 (34.4)	30/109 (27.5)
Adrenal	678/6673 (10.2)	NA	143/861 (16.6)	NA	NA	991/11 157 (8.9)	33/486 (6.8)	NA	NA	9/142 (6.3)	4/109 (3.7)
Brain	705/8796 (8.0)	NA	NA	NA	(9.8)	904/11 157 (8.1)	18/639 (2.8)	NA	NA	4/184 (2.2)	NA

Abbreviations: ccRCC, clear cell renal cell carcinoma; chrRCC, chromophobe renal cell carcinoma; NA, not applicable; pRCC, papillary renal cell carcinoma.

^a^ Not specific to ccRCC.

^b^ Not assessed at the time of first-line systemic therapy initiation.

^c^ Excluding retroperitoneum and mediastinum.

Of these prior studies, 2 retrospective observational studies conducted by Chandrasekar et al^13^ and Bianchi et al^12^ were least in keeping with our data, particularly when considering the reported rates of lung metastases. However, we note several key differences in study design that may explain these discrepancies. Namely, both studies were not specific to patients with ccRCC, used population-level administrative databases (Surveillance, Epidemiology and End Results and the Nationwide Inpatient Sample, respectively) reliant on diagnostic codes to identify metastatic sites, and did not specifically collect data at the time of initiating first-line systemic therapy for metastatic disease. Additionally, in the latter study,^12^ metastatic disease in the retroperitoneum and/or mediastinum was recorded separately from other lymph node metastases, which may at least partially explain the unusually low lymph node metastasis rate. We are encouraged that our data appear to align well with results from landmark prospective randomized clinical trials in ccRCC.^19,20,21^

In this study, patients with metastases to pleura, brain, liver, and bone were associated with the shortest median OS values. The latter 3 sites are well-known to be associated with inferior survival in mRCC,^16^ with the pleural site of metastasis representing a new finding. Conversely, although relatively infrequent, metastases to endocrine organs (pancreas, thyroid, adrenal) were noted to be associated with favorable OS outcomes. This result is consistent with prior data from smaller select cohorts.^14,25,26^ The prolonged OS times seen especially in patients with pancreas and thyroid metastases suggest favorable disease biology in this cohort of patients.

Indeed, elegant work from the TRACERx Renal Consortium has demonstrated that the clinical diversity of mRCC is underpinned by varied patterns of cancer evolution.^27^ Here, patients with pancreatic metastases were noted to have a significantly lower genome instability index compared with all other metastatic tissue sites, which may be related to their excellent clinical outcomes. Other groups have also reported on the unique biological profile of ccRCC that has metastasized to the pancreas.^28^

We were surprised to note the striking difference in OS for patients with metastatic chrRCC with metastases to the lungs (14 months) vs lymph nodes, bone and liver (26-28 months) (Table 2). This was especially notable given that patients with ccRCC or pRCC and metastases to the lungs in our study in fact had the longest median OS of these sites. These findings may be in part due to methodologic limitations, true differences in underlying disease biology, or both. From a study design perspective, patients with indolent chrRCC tumors involving the lungs may have been preferentially selected for local therapies (eg, metastasectomy), resulting in a bias toward patients with poorer prognosis starting systemic therapy. However, there are also data suggesting that patients with chrRCC and lung metastases may have unique disease biology compared with other sites of metastases. A recent study of 109 patients with metastatic chrRCC found that patients with pulmonary metastases had a higher rate of sarcomatoid differentiation (49%) than other sites of metastasis, including lymph nodes, bone, and liver (29%, 15% and 20%, respectively).^24^ To evaluate these findings, we performed a similar post-hoc exploratory analysis of our own cohort of patients with chrRCC and found similar results, with rates of sarcomatoid differentiation among patients with metastatic chrRCC and involvement of lung, lymph nodes, bone and liver to be 38%, 23%, 14% and 18%, respectively. Further efforts to investigate whether these preliminary and unexpected results are supported by data from other cohorts are recommended.

Brain metastases represent a specific site of interest because they are generally associated with a very poor prognosis, high degree of morbidity, require dedicated assessment (ie, are not captured on routine cross-sectional body imaging), and are relatively unresponsive to conventional systemic therapy. The 8% rate of brain involvement in ccRCC, as well as the lower rate of brain metastasis in pRCC and chrRCC noted in our study, are consistent with prior data.^12,13,29,30^ The very low rate of brain metastasis in pRCC (3%) and chrRCC (2%) suggests that screening asymptomatic patients for brain metastasis in these groups may be of less value than in patients with ccRCC. However, it should be noted that the actual rate of brain metastasis may be higher than what is reported here, as small or asymptomatic brain metastases may not have been detected if routine brain imaging was not performed.

Compared with our anticipated proportions of histologic variants based on data from localized RCC, our observed rates of pRCC (7%) and chrRCC (2%) were lower than expected. This finding may in part be explained by the previously demonstrated tendency of localized nonclear cell histologic variants to have a lower risk of recurrence and/or death following surgical resection, resulting in skewed proportions in our cohort consisting exclusively of patients with metastatic disease.^8,9,31^ In addition, the smaller proportions of patients with pRCC and chrRCC in our study could also in part be impacted by the fact that our cohort only included those who initiated systemic therapy. Patients with these histologic subtypes may have been less likely to receive systemic therapy as the efficacy of current standard of care treatments for mRCC is generally thought to be inferior in non–clear cell variants.^32^

### Strengths and Limitations

Strengths of our study include the very large sample size, international multicenter nature, consistency of results with previously reported figures, and detailed individual medical record review for each patient resulting in highly granular and reliable data. Furthermore, given the large sample size we were able to report on outcomes for rare cohorts including patients with pRCC and chrRCC, in addition to less common sites of metastasis in ccRCC. This is also the first study, to our knowledge, to compare rates of metastatic site involvement across histologic variants from a single cohort.

There are several important limitations to our study. First, the IMDC database only includes patients with mRCC who have started systemic treatment for metastatic disease. Thus, patients who have metastatic disease managed by alternate strategies, including active surveillance, metastasis-directed therapy (eg, metastasectomy, stereotactic body radiotherapy), and/or best supportive care alone, and never start systemic therapy are not captured. Therefore, there are likely minor groups of patients with relatively indolent disease (active surveillance and metastasis-directed therapy groups) or highly aggressive disease (best supportive care group) that were not included. In particular, this limitation could result in a bias toward underestimating the rates of highly favorable sites of metastasis (eg, pancreas, thyroid) and those with very poor prognosis (eg, brain). Second, there were some sites of metastasis that were not captured, including soft tissue (skin, muscle, adipose tissue) and parotid gland metastases, as well as perinephric locoregional recurrences. Third, although the number of sites of metastatic involvement were collected and presented, the total number of metastatic lesions per patient were not captured. Fourth, we were unable to separate pRCC into its known pathologically and genetically distinct subtypes, type 1 and type 2.^32^ Fifth, we were not able to report on which baseline staging investigations were performed in each patient. Given that the routine use of certain baseline staging investigations likely varies between practitioners and institutions (eg, cross-sectional brain imaging and dedicated bone imaging), there were likely inconsistencies in practice patterns that might have resulted in the underestimation of rates of metastatic site involvement.

## Conclusions

In this cohort study, sites of metastatic involvement differed on the basis of histologic subtype in mRCC and were associated with OS. These data highlight the clinical and biological variability between histologic subtypes of mRCC. Metastases to endocrine organs are infrequent but are associated with the longest median OS, whereas metastases to pleura, brain, liver, and bone are associated with poor OS. These benchmark values are useful for patient counseling and study design. Further research to characterize differences in immune, molecular, and genetic profiles between metastatic sites and histologic subtypes is encouraged.

## Notes

Supplement. **eTable 1.** Sites of Metastatic Involvement by Histology **eTable 2.** Number of Concurrently Involved Metastatic Sites **eFigure.** IMDC Risk Group by Site of Metastatic Involvement in ccRCC
