# Supplementary material for: Evaluation of Clear Cell, Papillary, and Chromophobe Renal Cell Carcinoma Metastasis Sites and Association With Survival
Source: JAMA Netw Open. 2021 Jan 21;4(1):e2021869. doi: 10.1001/jamanetworkopen.2020.21869 (PMC7821027; doi:10.1001/jamanetworkopen.2020.21869)
Supplement: Supplement. — eTable 1. Sites of Metastatic Involvement by Histology eTable 2. Number of Concurrently Involved Metastatic Sites eFigure. IMDC Risk Group by Site of Metastatic Involvement in ccRCC [file jamanetwopen-e2021869-s001.pdf]

Supplemental Online Content

Dudani S, de Velasco G, Wells JC, et al. Evaluation of clear cell, papillary, and chromophobe renal cell carcinoma metastasis sites and association with survival. *JAMA Netw Open*. 2021;4(1):e2021869. doi:10.1001/jamanetworkopen.2020.21869

eTable 1. Sites of Metastatic Involvement by Histology

eTable 2. Number of Concurrently Involved Metastatic Sites

eFigure. IMDC Risk Group by Site of Metastatic Involvement in ccRCC

This supplemental material has been provided by the authors to give readers additional information about their work.

© 2021 Dudani S et al. *JAMA Network Open.*

eTable 1: Sites of Metastatic Involvement by Histology

Metastatic Site	ccRCC (N=9252)	pRCC (N=667)	chrRCC (N=186)	P-value*
**Lung (95% CI)**	70% (69-71)	49% (45-53)	36% (29-44)	<0.01
**Lymph Nodes (95% CI)**	45% (44-46)	69% (65-73)	51% (43-58)	<0.01
**Bone (95% CI)**	32% (31-33)	29% (26-33)	33% (27-41)	0.26
**Liver (95% CI)**	18% (17-19)	22% (19-26)	34% (28-41)	<0.01
**Adrenal (95% CI)**	10% (9-11)	7% (5-9)	6% (3-12)	0.02
**Brain (95% CI)**	8% (7-9)	3% (2-4)	2% (0.6-5)	<0.01
**Pancreas (95% CI)**	5% (5-6)	1% (0.6-3)	2% (0.5-6)	<0.01
**Pleura (95% CI)**	4% (4-5)	3% (2-5)	0.7% (0-4)	0.03
**Peritoneum (95% CI)**	2% (1.5-2.2)	5% (3-8)	4% (1-9)	<0.01
**Spleen (95% CI)**	0.9% (0.6-1.1)	0.6% (0.1-2)	0.8% (0-4)	0.88
**Thyroid (95% CI)**	0.7% (0.5-1.0)	0.2% (0-1)	0% N/A	0.25
**Bowel (95% CI)**	0.7% (0.5-0.9)	0.2% (0-1)	1.5% (0.2-5)	0.24

ccRCC = Clear-cell renal-cell carcinoma; pRCC = Papillary renal-cell carcinoma; chrRCC = Chromophobe renal-cell carcinoma
*Chi-squared test across all three groups

© 2021 Dudani S et al. *JAMA Network Open.*

eTable 2: Number of Concurrently Involved Metastatic Sites

Metastatic Site	Number of Concurrently Involved Sites (IQR)
**Lung**	2 (1-3)
**Lymph Nodes**	2 (2-3)
**Bone**	2 (2-3)
**Liver**	3 (2-3)
**Adrenal**	3 (2-4)
**Brain**	3 (2-4)
**Pancreas**	3 (2-4)
**Pleura**	3 (2-4)
**Peritoneum**	3 (2-4)
**Spleen**	3 (3-4)
**Thyroid**	3 (2-4)
**Bowel**	2 (2-3)

IQR = Interquartile Range

© 2021 Dudani S et al. *JAMA Network Open.*

eFigure 1: IMDC Risk Group by Site of Metastatic Involvement in ccRCC

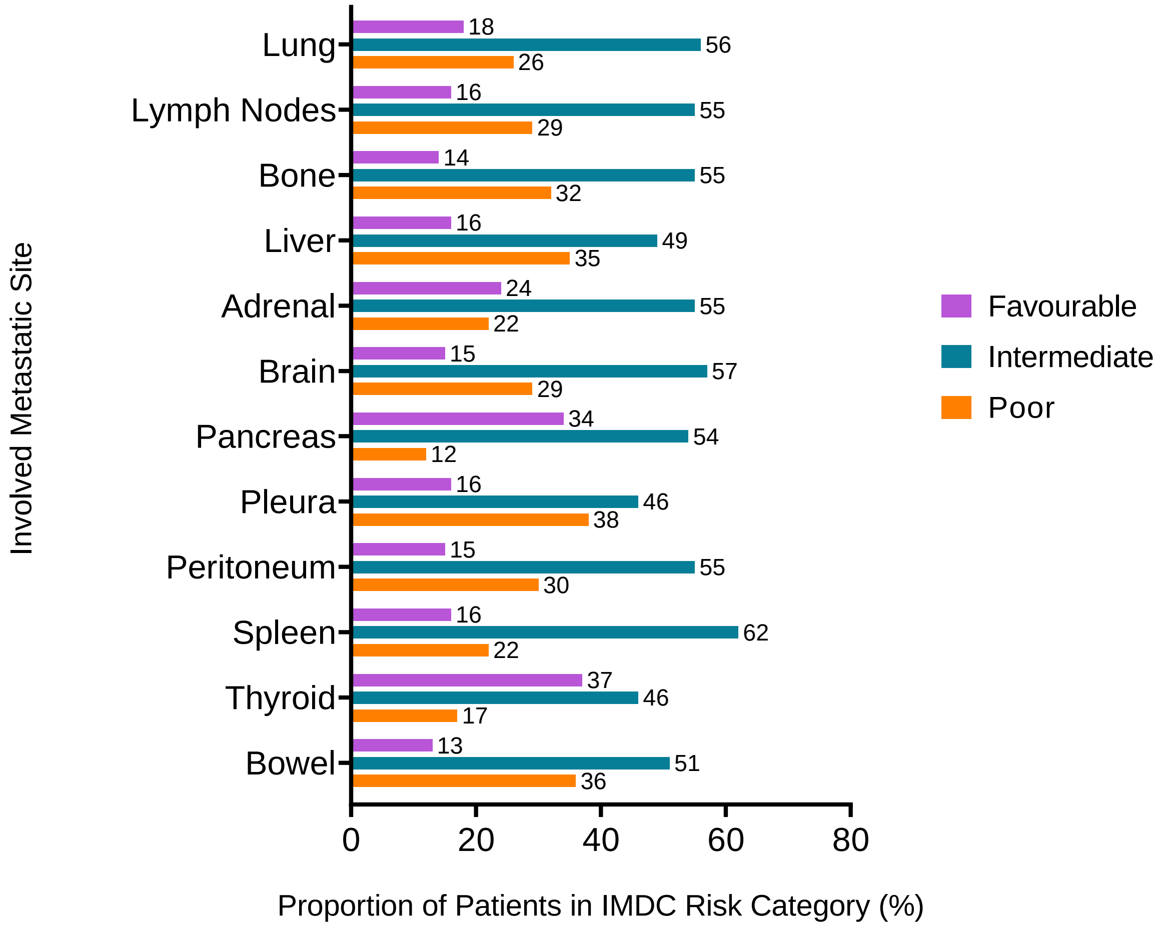

Caption: "*IMDC risk groups by sites of metastasis in patients with metastatic clear-cell renal-cell carcinoma. Highest rates of favourable-risk disease (>30%) noted in patients with metastases to thyroid and pancreas. Highest rates of poor-risk disease (>30%) noted in patients with metastases to bone, liver, pleura and bowel.*"

© 2021 Dudani S et al. *JAMA Network Open.*
